# Effects of the Comprehensive Cardiac Rehabilitation Program on Psychological Factors and Quality of Life among Coronary Heart Disease Patients

**DOI:** 10.5539/gjhs.v5n2p145

**Published:** 2012-12-24

**Authors:** Patrawut Intarakamhang, Ungsinun Intarakamhang

**Affiliations:** 1 Department of Physical Medicine and Rehabilitation, Phramongkutklao College of medicine and Hospital, Bangkok, Thailand; 2 Behavioral Science Research Institute, Srinakharinwirot University, Bangkok, Thailand

**Keywords:** Cardiac Rehabilitation Program, self-efficacy, self-regulation, self-care, quality of life, coronary Comprehensive heart disease

## Abstract

The Comprehensive Lifestyle Intervention, which integrates psychological and educational intervention, is a program to improve self-efficacy, self-regulation, self-care, body mass index and quality of life of the patients with coronary heart disease during early stages following hospitalization. The purpose of this study was to investigate the effects of the Comprehensive Cardiac Rehabilitation Program affecting psychological factors including self-efficacy, self-regulation, self-care, quality of life (QoL), and body mass index (BMI). This study was a quasi-experimental research with a repeated one group design. Eighty patients with coronary artery disease were recruited from either the Medicine or Surgical Ward at the Phramongkutklao Hospital where the patients joined the Comprehensive Cardiac Rehabilitation Program, which included attending exercising practice and receiving face-to-face counseling while being admitted to the hospital. Telephone counseling was thereafter performed one week after being discharged from the hospital, followed by undergoing individual or group counseling at the Cardiac Rehabilitation Clinic the following week. The follow-up period was performed within six weeks after hospitalization. Data was collected on two occasions before discharging from the hospital (pretest) and six weeks after (post-test) by using the self-efficacy, self-regulation, and self-care questionnaires, as well as the Short Form(SF) -36 (Thai version). The results indicated that by six weeks, 50%, 58.80%, 46.20%, and 72.50% of patients, respectively, had experienced increases with self-efficacy, self-regulation, self-care, and quality of life scores, while 12.50% of patients had decreased their body mass index in comparison with the pretest score. From the paired t-test, the self-efficacy, self-regulation and quality of life scores were statistically significant, having increased to the *p*<0.01 level; self-care was statistically significant, having increased to the *p*<0.05 level along with body mass index, which was statistically significant having experienced a decrease to the *p*<0.01 level.

## 1. Introduction

Cardiac rehabilitation is the art and science of integrating methods, techniques, and processes to assist patients, who have suffered from heart disease, in having better health and improving their quality of life. It is now widely accepted that it is important for patients with heart disease to be trained in cardiac rehabilitation. Cardiac rehabilitation is a process consisting of two major components: training exercises and behavioral change in order to reduce risk factors for cardiovascular diseases. In general, the program for restoration of the heart is respectively divided into four phases, including the inpatient phase, early outpatient phase, late outpatient phase and maintenance phase (Scottish Intercollegiate Guidelines Network, 2002).

According to Grace (2009), after studying 1,273 patients 65 and older with coronary artery difficulties during their early outpatient phase, it was found that older adults were already exercising at home (*p*=.001) and confident in their ability to self-manage their conditions (*p*=.003). Some barriers of cardiac rehabilitation were the perception of exercise as tiring or painful (*p*=.001), not knowing about cardiac rehabilitation (*p*=.001), lack of physician encouragement (*p*<.001), comorbidities such as diabetes, angina, and heart failure (p<.001), and perception that cardiac rehabilitation would not improve their health (*p*<.001) while distance (*p*=0.3), transportation problems (*p*=0.5) and cost (*p*=0.35) did not significantly differ.

The Phramongkutklao Hospital has been providing cardiac rehabilitation services since 1996 and has faced similar difficulties. Due to the latter, cardiac rehabilitation programs have shifted their program focus toward patient empowerment, enabling patients to practice by themselves at home, as well as the application of the Health Belief Model in restoring the heart, which focuses on the awareness of patients with regards to the risk, severity, benefits and barriers to practicing. The program involved psychological factors such as self-efficacy, which refers to a participant's belief in his/her own ability to maintain a healthy diet, engage in regular physical activity and stress management, perform self-regulation or make use of a planning process that activates and sustains positive and/or health related thoughts, take notes of their own behaviors and affects in order to attain the goal of cardiac rehabilitation, and perform self-care which refers to skills that focus healthy eating behavior (Ungsinun, 2011). These factors are known as “**factor** 3 **Self**.”

Many researchers have studied the techniques to raise the awareness of patients with coronary artery disease. For instance, Susan and colleagues (2009) found that calling patients at home after discharging them from the hospital every day for 6 weeks increased self-efficacy and quality of life. Sniehotta and associates (2003) conducted a randomized control trial study in patients with coronary artery disease. The techniques included the participation of the patients in a detailed action plan for treatment and keeping a journal in which a daily schedule for exercising and dieting was recorded. It was reported that patients had experienced a significant increase in self-regulation toward exercise and physical activity, in comparison with the control group. The researchers named that technique “**Innovative Psychological Intervention**,” which plays a crucial role in the achievement of self-regulation.

A number of studies have been performed and determined the role, emphasis and relation of these “3 Self” to health behavior. Roach (2003) integrated behavioral theories into a 12-week weight loss program and found that as self-efficacy improved, eating habits improved and weight loss was greater. Chang (2005) examined the self-control behaviors of diabetics and associated factors in 764 diabetic patients. Self-care behaviors included any of the following: taking medicine regularly, reducing weight, avoiding cigarettes or alcohol, exercising, practicing diet control, and maintaining a regular life style. The result showed that females in older age groups and those with a longer duration of diabetes took medicine regularly. Older age groups, individuals with a middle school education, and a longer duration of the disease had a higher chance of using any self-care method. Saksvig (2005) conducted a pretest/post-test, single-sample design of school-based diabetes prevention program in 122 students and found significant increases in dietary intention, dietary preference, knowledge, and dietary self-efficacy. Linde (2006) found relationships between self-efficacy beliefs, weight control behaviors, and weight change among 349 clients participating in a weight loss trial. Eating and exercise self-efficacy beliefs were strongly associated with corresponding weight loss behaviors. Riggs (2007) studied violence prevention programs that translate into an elementary school curriculum program for obesity prevention and the results showed significant changes in positive attitudes toward self-regulation of appetitive behavior. Smith (2010) studied an intervention combating childhood obesity with risk factors for Type 2 diabetes via a 12-week, two-session-per-week protocol, based on social cognitive theory and found significant intragroup improvement in the number of days per week of 60 or more minutes of voluntary physical activity. Significant changes in measures of both task self-efficacy (ß=.39), self-regulatory efficacy (ß=.44) and significant improvements in total cholesterol and body mass index were found. Finally, the method called “Comprehensive Lifestyle Intervention,” which is part of the Comprehensive Cardiac Rehabilitation program currently under review, covered four main areas (James, 2007), including a very low fat vegetarian diet, moderate aerobic exercise, stress management, and group support for the hospitalized patients as well as patients who were discharged from the hospital within 6-8 weeks (as it was seen as important in promoting self-care at home).”

In summary, Comprehensive Cardiac Rehabilitation program, exactly regulated by “3 Self”, consisted of both psychological and educational intervention and strongly improved any health behavioral modification such as weight loss program that indicated by body mass index and quality of life (as it reflected body and mind of the patient and was well known indicator of cardiac rehabilitation).

## 2. Objective

Due to the barriers of the patients to join outpatient or hospital based cardiac rehabilitation, the Comprehensive Cardiac Rehabilitation program would be flexibly performed in limited period about 6 weeks of inpatient to early outpatient phase. This led to the assumption of possible changes of “3 Self” and some outcomes of behavioral modification eq. quality of life and body mass index after 6 weeks. The objective of this study was to investigate the effects of the Comprehensive Cardiac Rehabilitation program on psychological factors which were composed of the following;

(a) self-efficacy,
(b) self-regulation,
(c) self-care,
(d) quality of life, and
(e) body mass index in patients with coronary artery disease.

## 3. Method

This study was a Quasi experimental design of the pretest and post-test single group.

### 3.1 Sample

Eighty coronary artery disease patients were recruited from the Phramongkutklao Hospital.

### 3.2 Inclusion Criteria

Participants with the following criteria were recruited to the program: 1) being able to participate in the entire program, 2) having no disease or mental health problems such as psychosis, neurosis mental retardation including moving difficulties or paralysis due to stroke, Parkinson, Alzheimer's disease and others, 3) having no chronic diseases in vital organs such as the lungs, liver, or kidneys, 4) no bedridden state or immobilization syndrome, and 5) a willingness to follow up at the Cardiac Rehabilitation Clinic at the Phramongkutklao Hospital after being discharged from the hospital for 2 months.

### 3.3 Setting

The participants were gathered from both private and community medical and surgical wards at the Phramongkutklao Hospital and followed up at Cardiac Rehabilitation Clinic, all of them were requested to have 5-6 times of life style intervention in Cardiac Rehabilitation Program within 6 weeks.

### 3.4 Measurements

The participants were evaluated twice: through pretest while they were hospitalized and post-test at Cardiac Rehabilitation Clinic which was 6 weeks after discharged from the hospital. There were two research instruments in this study. The first instrument was a 17-item questionnaire with Likert's scale which was used to assess influencing factors on health behavior for example exercises habit, eating habit, relaxation technique, narcotic and alcoholic avoidance, and adequacy of sleep. The responses were 1 to 4 that were not at all to definitely true. The 17 item questionnaire explored self-efficacy (5 items), self-regulation (5 items) and self-care (7 items) respectively (Ungsinun, 2011). Its Cronbach's alpha coefficient was between 0.73 and 0.85 and item correlations were between 0.29 and 0.76. The second instrument was the Short Form(SF)-36 (Thai version) to measure quality of life which covered eight subscales, including vitality, physical functioning, bodily pain, general health perceptions, physical role functioning, emotional role functioning, social role functioning, and mental health with a rating scale of 2-6 according to each subscales.

### 3.5 Intervention

1) Preparation phase

The workshops were set for trainers and counselors which are displayed in Table 1. The handbook for cardiac rehabilitation, as well as notebooks for recording eating habits and exercising (Exercise and Diet Log book) were also designed and developed.

**Table 1:** Workshop schedule for trainers and counselors

Day 1	Time (Mins)	Topics
	60	Basic exercise physiology for cardiac rehabilitation
	90	Inpatient cardiac rehabilitation
	30	Questions and answers

Day 2	30	Practicing inpatient cardiac rehabilitation
	30	Principles of 3Self, PROMISE (Positive reinforcement: P, Result Based Management-RBM: R, Optimism: O, Motivation: M, Individual Or Clients-Center Approach: I, Self-esteem: Se)
	30	Steps of behavioral changes, SF36
	30	Using-application SF-36
	30	Bedside counseling for: Diet control, stress control, physical activity Discharge planning
	30	Data collection and indicators of the project Follow-up calls and follow up at rehabilitation at OPD
	30	Questions and answers

**Figure 1:**
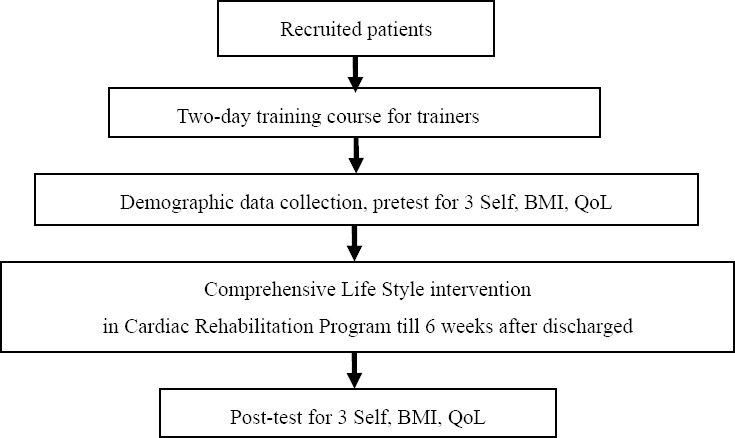
Flowchart of the comprehensive life style intervention

2) Intervention phase (Figure 1)

2.1) providing bedside training focused toward exercising at least twice or more under the coaching of trainers.

2.2) giving counseling for both patients and caregivers, in which the topics of simple diet control with low fat, low salt, and less sweets, stress reduction, and recording in the Exercise and Diet Log book were covered. Motivating bedside exercising was then performed.

2.3) delivering health advice to the participants via a telephone call one week after being discharged from the hospital.

2.4) setting individual or group counseling of 30-60 minutes in length at the Cardiac Rehabilitation Clinic two weeks after discharging from the hospital.

2.5) re-evaluating Exercise and Diet Log book recordings and a post-test 6 weeks after leaving the hospital.

### 3.6 Statistical Analysis

The demographic data were analyzed by using descriptive statistics (i.e., frequency, percentage, mean, and standard deviation). The Paired t-test was used to compare the pretest and post-test score of the psychological factors (self-efficacy, self-regulation, self-care, body mass index, and quality of life).

## 4. Results

The sample consisted of 80 patients with coronary artery disease, in which 66 (62.66%) participants were male and 14 (37.34%) participants were female: 42 participants were aged 60 years or older (52.5%), 26 were in the age group of 50-59 years (32.5%), 28 participants had no education (35.0%) whereas 24 participants completed primary school (30%); 56 (70%) participants were covered by the Civil Servant Medical Benefit Scheme (CSMBS) and 13 (16.25%) by the universal coverage scheme. The risk factors were comprised of hypertension (83.75%), hypercholesterolemia (67.5%), smoking (61.25%), lack of exercise (55%), diabetes (53.75 %), obesity (50%), increased body mass index (40%), and stress and genetic (27.5%). Sixty-five (81.25%) patients had Coronary Bypass Graft surgery (CABG), fourteen (17.5%) received cardiac catheterization treatment, and one pursued only oral medications.

The findings of the post-test are shown in Table 2 indicated that fifty-nine (73.75%) patients had blood pressure less than 140/90 mmHg and every patients did not need additional anti-hypertensive medicine. Forty-five out of forty-nine (91.83%) smokers quit smoking. Fifty-seven (71.25%) patients performed walking exercise for at least 20 minutes five days a week throughout the 6 weeks and every patients can exercise safely. Also, 73 (91.25%) patients indicated high satisfaction in participating in the program. Additionally, in Table 3, 40 (50%), 47 (58.80%), 37 (46.20%), and 58 (72.50%) of patients had increased self-efficacy, self-regulation, self-care, and quality of life, correspondingly, while 10 (12.50%) patients had decreased body mass index. Furthermore, in table 4, the score of self-efficacy, self-regulation, and quality of life of patients with coronary artery disease after the program were statistically significant and higher than those prior to the intervention (*p*=.01). The score of self-care after the program was statistically significantly higher than that prior to the intervention (*p*=.05). The score of BMI after the program was also statistically significantly lower than that prior to the intervention (*p*=.01).

**Table 2:** Changes of variables at the end of the 6 weeks

Variables	Number (%) (male=62.66%, female=37.34%)
Blood pressure <140/90 mmHg after the program	59 (73.75)
Patients did not need additional anti-hypertensive drugs	80 (100)
Smoking cessation	45 from all 49 male (91.83)
Being able to do home walking exercise at least 20 minutes for 5 days per week	57 (71.25)
Patients can exercise safely at home	80 (100)
High level of satisfaction toward the program	73 (91.25)

**Table 3:** Number and percentage of patients with 3 Self, QoL and BMI increased, decreased, and unchanged after the Cardiac Rehabilitation Program

Variables N = 80	Increased		Unchanged		Decreased	

	Number (Person)	%	Number (Person)	%	Number (Person)	%
Self-efficacy	40	50.00	16	20.00	24	30.00
Self-regulation	47	58.80	17	21.20	16	20.00
Self-care	37	46.20	29	36.20	14	17.50
BMI	14	17.50	56	70.00	10	12.50
QoL	58	72.50	1	1.20	19	23.80

**Table 4:** Comparison of 3 Self, QoL, and BMI before and after the Cardiac Rehabilitation Program

Variables N=80	Before		After		M.D.	*t*	*p*

	x̄	S.D.	x̄	S.D.			
Self-efficacy	14.0	2.91	15.76	2.03	0.862	3.014	0.005*
Self-regulation	15.78	2.12	14.47	3.13	1.312	4.076	0.000*
Self-care	21.90	3.81	22.71	2.59	0.812	1.982	0.050*
BMI	25.22	3.21	24.82	3.10	-0.396	3.430	0.001*
QoL	52.17	16.89	62.97	19.31	10.79	4.800	0.000*

* *p* value at 0.05

## 5. Discussion

81.25% of the patients with coronary artery disease received surgery Coronary Bypass Graft surgery (CABG) and are always considered as elective cases which have clinical practice guidelines to follow. Every patient especially needs to join the inpatient cardiac rehabilitation program.

Table 2 indicates that all the variables had affected more than 70% of the patients by the end of week 6. Variables that were achieved up to 100% were patients do not need additional anti-hypertensive drug and performing walking exercise safely. For variables such as controlling blood pressure, smoke cessation, and regular walking related to the Health Belief Model which describes the patient's internal psychological factors, including self-efficacy, self-regulation which results in patients’ self-care.

Lower body mass index (Tables 3 and 4) is the result of dietary control and regular exercise, which patients gained knowledge and skills for from the Comprehensive Cardiac Rehabilitation Program or Comprehensive Lifestyle Intervention. However, this change might be influenced by the surgery which caused patients to eat less and lose weight.

Quality of life (Table 3 and 4) increased in SF-36 questionnaires for all eight subscales which can be re-combined into four categories: physical health, psychological health, social relationship and environment, or it may simply be the physical and mental aspects.

Table 4 shows that all variables improved significantly, in particular psychological factors and **3 Self**, which is congruent with Roach (2003), Saksvig (2005), Linde (2006), Riggs (2007) and Smith (2010) who studied all factors 3 self in weight loss, weight control programs for children, teenagers, the obese and those susceptible to diabetes. Chang (2005) also found that older adults with diabetes had better self-care behavior than younger patients, which is similar to this study in term of the age of participants.

**Figure 2:**
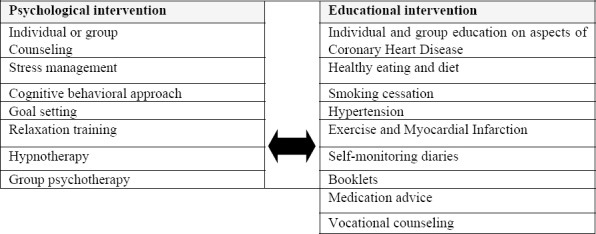
Various techniques of psychological intervention and educational intervention

Linden (1996) and Dusseldorp (1999) discussed that psychological intervention consisted of a variety of techniques (Figure 2). The techniques used in the Comprehensive Cardiac Rehabilitation Program included individual and group counseling, stress management and relaxation and goal setting while the educational interventions also include other various techniques (Figure 2). The Comprehensive Cardiac Rehabilitation Program in this study adopted various topics including individual and group education for coronary heart disease, healthy eating and diet control, smoking cessation, hypertension control, self-monitoring diaries and log books, booklets distribution and cardiac medication.

Dusseldorp (1999) conducted a meta-analysis of 8,988 patients in 37 trials and found that cardiac rehabilitation programs including psychological and/or educational interventions resulted in a 34% reduction in cardiac mortality and a 29% reduction in recurrent myocardial infarction at 1-10 years follow up.

As a result, the Comprehensive Lifestyle Intervention or Comprehensive Cardiac Rehabilitation Program in this study significantly improved all variables in agreement with the study of Ungsinun (2011), which found that the risk of metabolic syndrome of 3,414 people from non-government organizations (NGOs), after engaging in the Health Behavioral Modification Program (HBMP) five times in the previous 4-7 months with 3 self-behavior variables including self-efficacy, self-regulation and self-care, are significantly higher and decreased body mass index significantly (*p*<.001). The similar study of Ungsinun (2012) in 4,649 people who are at risk of metabolic syndrome from 17 hospital, after engaging in the Health Behavioral Modification Program (HBMP) for 5-7 months found that the same 3 self- behavior variables increased significantly and BMI decreased significantly (*p*<.005). Janssen (2012) performed meta-analysis of 23 trials involving 11,085 randomized patients to determine the efficacy of lifestyle modification programs for coronary heart disease patients developed over the last decade (1999-2009) and the results showed lifestyle modification programs positively affected risk factors and related lifestyle behaviors at post treatment (mean=10.2 months), and some of these benefits were maintained at long-term follow ups (mean=33.7 months). Improvements in dietary and exercise behavior were greater for programs incorporating all four self-regulation techniques (i.e., goal setting, self-monitoring, planning, and feedback techniques) compared to interventions that included none of these techniques.

Recommendations for future research are: 1) to study the relationship between variables of **3 Self** with quality of life, body mass index, and the ability to perform additional exercise, 2) to monitor these variations in the long term for 3, 6, 12 months after hospitalization in order to reflect the performance of health behavior, 3) to study the application of theories and techniques of psychological intervention and educational intervention such as cognitive behavioral approach in the cognitive behavior of patients, the Health Belief Model in the stimulation and promotion of patients’ self-efficacy, motivation intervention to establish intrinsic motivation, and the commitment of goal setting in cardiac rehabilitation between patients and health personnel.

## 6. Conclusion

This Comprehensive Cardiac Rehabilitation Program or Comprehensive Lifestyle Intervention for patients with coronary artery disease including psychological and education interventions had significantly improved self-efficacy, self-regulation and self-care, body mass index, and quality of life within 6 week after hospitalization (early outpatient phase of cardiac rehabilitation).
